# Experiencing Independence: Perspectives from Autistic Adults

**DOI:** 10.1007/s10803-025-06812-0

**Published:** 2025-04-03

**Authors:** Piyali Bhattacharya, Rose J Matthews, Rae Field, Hannah Heath, Kate A. Woodcock, Andrew D. R. Surtees

**Affiliations:** 1 School of Psychology, University of Birmingham, Birmingham, UK; 2 Independent Researcher, Durham, UK

**Keywords:** Independence, Autistic adults, Lived experience, Empowerment, Thematic analysis, Qualitative study

## Abstract

Autism diagnostic criteria relate to difficulties in functioning across multiple developmental domains, that often impact on a person’s independence. There are different ways to conceptualise and exercise independence, but no previous studies have questioned how autistic adults do so. This qualitative study aimed to understand how autistic adults define and experience independence. It was designed to further our understanding of how autistic adults account for and use coping strategies to overcome barriers to independent living and navigate their way to their desired level of independence. Twelve semi-structured interviews were conducted with autistic adults living in the United Kingdom. The data were analysed using reflexive thematic analysis. The researchers generated three major themes. The first theme ‘Independence is “not a one-size-fits-all”’ highlights that there is no one definition or concept of independence for autistic people; these are relative and uniquely individual. The second theme ‘“Being autistic has its setbacks” in a neurotypical world’ describes the obstacles encountered by autistic adults seeking independence within a society that favours neurotypical norms. The third theme ‘Finding ways of making it work’ captures strategies autistic adults use in achieving or maintaining independence. The findings from this study provide a foundation for future research exploring domains of independence for autistic adults. The perceived desirability of achieving different degrees of in(ter)dependence and the fluctuating nature of self-sufficiency are explored through lived experience. Increasing understanding of barriers and challenges to independence has the potential to empower autistic adults and improve services and support.

**Supplementary Information:**

The online version contains supplementary material available at 10.1007/s10803-025-06812-0.

By conventional metrics, autistic people often experience lower levels of independence than their neurotypical peers (McLeod et al., 2019, 2021; McQuaid et al., 2022). Such metrics are typically reliant on definitions of independence based on the measurable amount of support a person requires. With this in mind, the focus of research has often been on the nature of transitions to adulthood, with employment being seen as a key factor in allowing someone to live without substantial support. In the United Kingdom (UK), only 29% of autistic adults are in some kind of paid employment, while 76% of autistic adults live with their parents (Ayoubkhani et al., 2021). Autistic people are also less likely to possess their own homes, as compared to other neurodivergent and disabled groups of people (Ayoubkhani et al., 2021). Despite recent advancements in our understanding of autism, there is little research about how autistic adults define and experience independence. In developing our understanding of autism in adulthood, exploring what being independent means to autistic people, and investigating the factors that impact their ability to achieve this, is extremely important. It will allow us to understand how, when and where autistic people want to exercise their independence. Further, it will help optimise environments, and provide support and adjustments for autistic people who want and need them (Thompson et al., 2018).

Independence has predominantly been operationalised from a neurotypical perspective (Henninger & Taylor, 2013; Hume et al., 2014; Hume & Odom, 2007; Wehmeyer, 2000; Zimmer-Gembeck & Collins, 2006). The most common approach to defining independence revolves around the ability to complete functional aspects of daily living, such as cooking, bathing, etc. (Hume et al., 2014; Hume & Odom, 2007; Wise et al., 2020). However, this is not the only way that people consider themselves to be independent. Having a job, being economically self-supporting, and living away from one’s parents may also be seen as definitional aspects of independence (Cunningham & Diversi, 2013).

Several studies have suggested that autistic people experience particular challenges in achieving conventionally-defined independence, relating to functional (Gotham et al., 2015; Lin et al., 2012) and psychological (Cooper et al., 2017; Nguyen et al., 2020) aspects of independence. Why autistic people experience lower independence may be explained by a range of contributory factors (Hong et al., 2016; Hume et al., 2014). These include the core characteristics of autism (Howlin, 2005; Howlin et al., 2000; Hume et al., 2014), or co-occurring mental health conditions, such as anxiety and depression (Lai et al., 2019). Cognitive challenges relating to executive functioning can also impact goal-setting, planning, memory, task-switching, and flexibility, all of which can all be vital for independence (Hume et al., 2014; Ozonoff & Schetter, 2007). Furthermore, structural and environmental factors, such as barriers to employment, lack of workplace support (Lorenz et al., 2016), inadequate training (Kim, 2024), and inaccessible leisure and public services (Brewster & Coleyshaw, 2011), due to sensory and environmental challenges (Doherty et al., 2023; MacLennan et al., 2023) have resulted in significant inequalities. These challenges also lead to adverse outcomes, such as restricted access to services and support, social isolation, economic disadvantage, financial precarity, and poor health (Pellicano et al., 2022).

Despite the significance of independence for autistic people, service providers, and policymakers, no studies have explored how autistic people conceptualise and experience their own independence. A few studies have, however, examined autistic people’s perspectives on the factors that influence their independence. These studies have primarily defined independence from the perspective of independent living - living on one’s own, the nature of one’s physical living arrangements, and specific functional abilities (Al Ansari et al., 2024; Ghanouni et al., 2021). Ghanouni et al. (2021) investigated independent living in autistic adults in Canada, interviewing 13 autistic adults and six parents. The authors concluded that autistic adults’ ability to manage their finances and live within integrated communities and housing, whilst managing their autistic characteristics contributed to independent living (Ghanouni et al., 2021). However, this paper focused solely on the functional aspects of independence. A more recent qualitative study explored the independent living arrangements of 11 Australian autistic adults when considering the moving out (transitioning) phase, and their challenges related to that phase (Al Ansari et al., 2024). They found that personal space with autonomy and control, and the impact of diagnosis on accommodation choice and freedom, contributed to independent living. Both these recent qualitative studies (Al Ansari et al., 2024; Ghanouni et al., 2021) conflated independent living (process of home-leaving) defined by physical, functional, and logistical aspects like housing, finances, and living arrangements, primarily equating it with the capacity to live without parental support. Moreover, both studies focussed on pre-determined, neurotypical descriptions of independence, primarily based on an adaptive functioning^1^ framework. In doing so, they neglected alternative autistic perspectives, which are essential to a holistic understanding of the subject.

It is critical to distinguish between “independence” and “independent living,” as these concepts, while interconnected, are not synonymous. Although these concepts are often seen within a normative developmental trajectory associated with entering adulthood, autistic people may perceive independence in different ways, irrespective of living arrangements. A qualitative study that actively seeks to understand how autistic people conceptualise and experience their independence is crucial. Such research can challenge the traditional, neurotypical narrative that often defines independence in narrow, standardised terms. It may also provide a more inclusive understanding of what independence means to autistic adults, better reflecting their values, strengths, and diverse life circumstances.

To eliminate barriers and support autistic adults towards desired levels of independence, we need not only to understand autistic perspectives on independence but also to understand autistic perspectives on coping mechanisms. Several qualitative (Dachez & Ndobo, 2018; Ghanouni & Quirke, 2023) and quantitative (Muniandy et al., 2022) studies have been conducted to examine the coping mechanisms autistic adults use to navigate life independently. Special interests, militancy, diagnosis, seeking support from neurotypical friends, seeking support from animals, normalisation, intellectualisation, and humour (Dachez & Ndobo, 2018), seeking emotional support, engaging in recreation and leisure, adopting technology to adjust routines (Ghanouni & Quirke, 2023), social camouflaging (Hull et al., 2017; Schneid & Raz, 2020) are some of the strategies that autistic adults have identified using to navigate their way towards independence. Again, though, studies have tended to explore coping mechanisms through neurotypical frameworks and expectations of adaptive and social functioning, rather than investigating how coping mechanisms allow autistic people to achieve independence on their own terms.

Independence is a complex concept, operationalised in myriad ways (Hume et al., 2014). So far, studies have conceptualised independence from a neurotypical perspective, and most evidence has come from non-autistic people, such as service providers, researchers, and parents (Gillespie-Lynch et al., 2017). It is crucial to involve autistic people in discussions about how they perceive and define independence rather than creating a one-size-fits-all definition of independence based on societal norms and expectations that may differ from their aspirations (Botha & Cage, 2022; Fletcher-Watson et al., 2019). This qualitative study is designed to gain an in-depth understanding of how independence is conceptualised by autistic adults in the UK.

The research questions of this study were:

1. How do autistic adults in the UK make sense of experiencing independence?
2. How do autistic adults in the UK develop coping strategies to tackle the barriers to independence and navigate their way towards independence?

## Methodology

The study employed a qualitative research design using Reflexive Thematic Analysis, within an “iterative-inductive approach” (O’Reilly, 2012, p. 30) to explore and evaluate themes through semi-structured interviews. This approach emphasises ‘fluidity and flexibility’ in the research process (Braun & Clarke, 2019). This was attempted by keeping in mind that the autistic adults were expert informants and guides on their own unique worlds and that their perspectives should be prominent throughout the study. We employed a critical realist philosophical stance. Many recent qualitative studies with autistic people have adopted this approach (Botha et al., 2022a, b; Raymaker et al., 2023) and generated meaningful interpretations. In this study, critical realism focused on the lived experiences of participants in relation to independence, whilst also incorporating the cultural and social factors and insights that form the basis of their narratives. Autistic people are frequently discriminated against and marginalised (DePape & Lindsay, 2016; Radulski, 2022), foregrounding their perspectives was a way of acknowledging the epistemic injustice they experience.

### Recruitment & Participants

Twelve autistic adults were recruited through responses made to adverts shared with a local autism research database, local support groups, and charities. As 10–20 is a recommended sample size for a medium TA project (Clarke, 2013), we implemented a reflexive approach to recruitment. All eligible participants who responded were recruited on a first come, first served basis. We ceased recruitment when we felt there were enough data to adequately address the research question (Braun & Clarke, 2021b). This decision was guided by the concept of information power, which suggests that the more relevant and richer the data, the fewer participants are needed to answer the research question (Malterud et al., 2016). The process was iterative as throughout the recruitment process, we continuously reviewed the data, assessing its depth and relevance in relation to the study’s aims. As we analysed the data, we found that the information being gathered was sufficiently focused and directly addressed the research question, without the need for additional participants.

Participants were eligible if they were diagnosed with an autism by a certified professional, were aged between 18 and 50 years, and had the capacity to give informed consent, in accordance with the Code of Human Research Ethics (*Code of Ethics and Conduct*, 2021). Applicants were not eligible if they had severe and enduring co-occurring conditions, including intellectual disability, severe mental illness (e.g., Schizophrenia, Bipolar Disorder, Personality Disorder, etc.), physical health problems (e.g., cancer, TB, etc.), or sensory impairments (e.g., deafness or blindness). This was due to the significant impact that these conditions would be likely to have on independence, contributing to the complexity of understanding the impact of autism on independence (Bishop-Fitzpatrick & Rubenstein, 2019; Lin & Huang, 2019; Mason et al., 2018).

Participants were UK residents, aged 24 to 48 years (mean age = 32.41 years), seven were male. The average age of diagnosis of autism was 21.58 years; see Table 1 for the detailed demographics of the participants.

**Table 1:** Demographic characteristics of the sample

Characteristic	Full Sample = 12
Age years; M, (SD), [Range]	32.5 years, (8.2), [24–48]
Gender, n (%)	
Female	3 (25%)
Male	7 (58%)
Nonbinary	2 (17%)
Marital Status, n (%)	
Married	2 (17%)
Unmarried	10 (83%)
Living arrangement, n (%)	
Living with parents	4 (33%)
Living with partner/spouse	3 (25%)
Living alone	3 (25%)
Living with housemates	2 (17%)
Employment Status, n (%)	
Employed	11 (92%)
Not Employed	1 (8%)
Age of Autism diagnosis; M, (SD), [Range]	21.5 years, (12.3), [5–46]
Diagnosed in childhood/adolescence	5 (42%)
Diagnosed in adulthood	7 (58%)
Co-occurring mental health conditions, n	
Depression	7
Anxiety	6
Attention Deficit Hyperactive Disorder	4
Obsessive Compulsive Disorder	2
Agoraphobia	1
Insomnia	1

### Ethics

This study was reviewed and approved by the Science, Technology, Engineering, and Mathematics committee. All participants were assigned a unique code and transcripts were anonymised. Prior to interview, participants were informed about the purpose and process of the study, while assuring their voluntary participation. All data were stored in accordance with university regulations.

### Data Collection

Interviews were semi-structured, facilitating in-depth discussion between the researcher and the interviewee (Adams, 2015). The schedule (see supplementary materials) was designed by the primary researcher (PB) and revised and improved after consultation and discussion with other members of the research team (AS & KW). The questions were open-ended and focused mainly on autistic adults’ perceptions of experiencing independence, lived experience of independence in their day-to-day life, major hurdles and barriers that stood in their way to independence, and strategies they used to overcome these to experience independence.

Potential participants were provided with an overview of the study, including an explanation of its objectives. Those who were interested in participating were invited to contact the lead researcher (PB) and asked to sign an informed consent form. Interviews for the study were completed between November 2022 and March 2023 by PB, using a secure institutional Zoom licence. Good practice in discussing potentially sensitive topics in interviews was followed (Silverio et al., 2022), including establishing rapport through the interviewer introducing themselves and engaging in basic self-disclosure. Participants were not provided with an operational definition of independence at the time of enrolment. This was a deliberate methodological decision to avoid imposing a predefined framework that could bias participants’ conceptualisation of independence. Instead, participants were given freedom to express their own perspectives on what independence meant to them, ensuring that their experiences and viewpoints were captured authentically.

Care was taken to follow the participant’s lead during the interview while navigating the conversation using the semi-structured interview question guide. Participants were not pressed to answer any questions and instead were encouraged to talk about their experiences. Additional open-ended questions were used to prompt more information from participants when deemed necessary (Adams, 2015). Throughout the interview, PB took detailed notes, capturing thoughts, feelings, impressions, and key responses from the participants. These notes were used to complement the audio recordings and to provide additional context for later data analysis (Trainor & Bundon, 2021). The interviews were transcribed by RF and underwent a quality check by PB to ensure accuracy. This was done by reading the transcriptions alongside listening to the original recording and making changes as appropriate. These interviews lasted half an hour to one and a half hours (Mean = 1 h). Participants were compensated with a £10 Amazon gift voucher.

### Data Analysis

We analysed our data using Reflexive Thematic Analysis (RTA). RTA was deemed appropriate for this study because it is data-driven, adaptable, and sensitive to examining complex meanings within data, which is crucial for issues that have not yet been well investigated (Braun & Clarke, 2019, 2021b). RTA’s flexibility offered the possibility for an inductively developed analysis which helped us to capture semantic and latent meanings and offered both descriptive and interpretative accounts of the data (Braun & Clarke, 2021b). RTA is not tied to a theoretical framework and thus offers a flexible approach in exploring differing autistic perspectives on independence, thereby allowing for an experiential analysis of the patterns of meaning across the data (Braun & Clarke, 2019, 2022).

This approach aimed to explore recurring patterns within the data, bringing attention to significant facets of the concept of independence seen from autistic adults’ perspectives. We followed a 6-step process in analysing the data using RTA, as outlined by Braun and Clarke (2019). This was done by engaging and familiarising ourselves with the data, taking a conscious, thoughtful, reflective, and intentional approach. PB initially coded the transcripts. Line-by-line coding was conducted which generated semantic (descriptive, surface-level) and latent (underlying, implicit) hand-written codes. After PB completed coding all the transcripts, RM reviewed both the code list and the text by re-reading the transcripts, focusing on the micro-level meaning which helped to develop “fine-grained codes” (Braun & Clarke, 20062021a, p. 42). During this review, RM focused on the micro-level meaning of the participants’ responses, paying close attention to subtle nuances, patterns, and underlying themes that may have been missed in the first round of coding. This careful and detailed examination allowed RM to “fine-grained codes” (Braun & Clarke, 2021a, p. 42), capturing more precise distinctions in meaning and refining the overall coding structure.

PB subsequently created the initial themes through an inductive approach, by clustering overarching codes according to the meaning or underlying central-organising concepts. This method involved identifying patterns in the data and clustering codes that reflected similar ideas, allowing overarching themes to be generated from the content. Each theme and specific segment of coded text identified by PB underwent a thorough review and refinement process during coding discussion meetings with RM and RF. This was done by visually mapping the themes and sub-themes into a spreadsheet which aided in the refinement of the boundaries between themes and connections within the sub-themes. Full consensus on coding and the development of themes/subthemes was reached among PB, RM, and RF during these discussions. We encountered challenges during the process, due to differing perspectives rooted in neurodevelopmental experiences. These challenges mainly centred on language and terminology, as certain terms were interpreted differently by autistic researchers. In some cases, there were conceptual disagreements as well, particularly around the notion of “achieving independence” being framed as a “triumph”. The autistic researchers expressed discomfort with this idea, as it implied that dependence was inherently negative, which conflicted with their views on interdependence and support within the neurodivergent community. This difference in perspective required careful navigation and collaboration to ensure that the final themes were inclusive and reflective of both neurotypical and neurodivergent viewpoints. The themes and subthemes were then presented to AS and KW for additional discussion and refinement.

### Community Involvement

It is becoming more widely acknowledged that autistic people are knowledgeable experts on the autistic experience and skilled researchers (Gillespie-Lynch et al., 2017; Grant & Kara, 2021). The phrase “Nothing about us without us” (Hoekstra et al., 2018, p. 40) is frequently used to describe how autistic people have the right to be involved in decisions made about public policy and services that affect them. Two autistic researchers contributed to co-creating the themes and sub-themes that have offered valuable firsthand insights into the lived experiences of autistic participants, ensuring that the themes were more accurate, relevant, and reflective of their unique perspectives. Their involvement helped identify subtle nuances and meanings that neurotypical researchers might overlook, enhancing interpretive rigor and leading to a more inclusive and comprehensive data interpretation (Botha & Cage, 2022). While autistic co-researchers actively participated in data analysis and subsequent stages, their involvement began too late to influence the initial research design and data collection process. If autistic collaborators had been involved earlier, the research would have been more inclusive and robust (Hobson et al., 2023; Kaplan-Kahn & Caplan, 2023; Stark et al., 2021).

### Reflexivity & Embodiment

Four authors of this study are non-autistic, and the remaining two are autistic. Five authors have a background in Psychology, with four specialising in autism research. PB identifies as an Indian, heterosexual, neurotypical female. AS, KW, and HH are Caucasian, from the UK, heterosexual, and serving as senior researchers with 10–20 years of research experience. RM is white British, non-binary, neuroqueer, and an experienced autistic autism researcher, and RF is a postgraduate female autistic researcher. The neurodiversity viewpoint shared by all authors acknowledges that although autistic people experience unique ways of seeing the world, these are not signs of weakness (deficits). The diverse backgrounds of the team - culturally, neurodevelopmentally, and in terms of gender identity - enhanced the reflexivity of the research process by challenging potential biases and ensuring that different viewpoints were actively considered and incorporated into the analysis. This reflexive approach strengthened the project’s commitment to capturing the nuanced experiences of autistic people, particularly in how they define and experience independence.

## Results

The reflexive thematic analysis of twelve interviews led to the creation of three primary themes:

*Theme 1 - Independence is Not a one-size-fits-all* depicts qualitative differences in how autistic adults understand independence in a multitude of ways.

*Theme 2 - Being autistic has its setbacks in a neurotypical world* depicts how autistic people experience a range of difficulties and challenges in achieving independence in a predominantly neurotypical society.

*Theme 3 - Finding ways of making it work* charts how autistic adults navigate or accommodate barriers to independence.

**Fig. 1:**
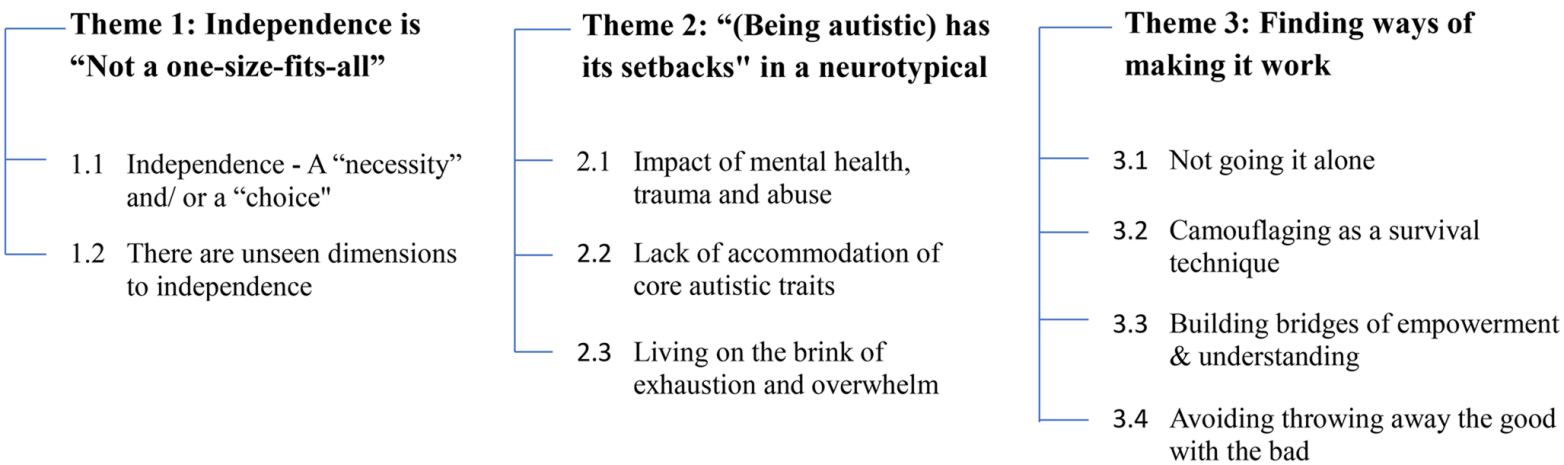
Participants’ perspectives on experiencing independence (Themes & Sub-themes)

The holistic concepts of the themes presented incorporate the following sub-themes, so the figure below (Fig. 1) is presented as a visual summary of the main elements of each theme.

### Theme 1: Independence is “not a one-size-fits-all”

There is no one definition of independence for autistic people as participant highlighted there is *“no one size fits all”* [P2]. Participants described independence as a relative concept, not an absolute one, reporting that everyone’s definition will be different depending on *“context*,* circumstance*,* life choices*,* life changes*,* expectancy”* [P2], encompassing diverse degrees and interpretations. The sub-themes reflect this variability.

#### Independence - A “necessity” and/ or a “choice”

Participants regarded independence as important for all autistic people, and some participants had felt compelled to become independent out of *“necessity”* [P9], due to their personal circumstances.

*I was thrown into the independence… I had to learn to do it*,* whereas if I had all the family support*,* would I be as independent as I am now? Probably not*,* because you’d stay in your comfort zone.* [P9]

Parents often played a pivotal role in the development of independence by determining whether participants had the option of carrying on living at home and whether they jumped willingly into independence or felt pushed. Some participants reported being forced into independence by perceived neglectful parental relationships.

*I basically parented my mum from the age of 8. And then my sister and my- and then my half-sisters. So*,* she- yeah*,* I didn’t really have a parent. My dad wasn’t really around. Um*,* my parents split up when I was- well*,* they divorced when I was 8.* [P4]

Other participants reported pushing back against parents who they felt had undermined their independence by being over-protective. They recounted a sense of having been *“infantilised and underestimated”* [P5] for a significant part of their life. In one instance, financial exploitation by their mother had exacerbated the participant’s desire for independence. In such circumstances, achieving independence was viewed as a *“necessity”* [P9] rather than a *“choice”* [P9], and driven by a deep-seated need to validate their capabilities and assert their autonomy.

Conversely, for other participants, independence was seen as a matter of choice, something they could pursue or defer with no fixed timescale. In these interviews, we see the complex interplay between an internal desire for independence and external limitations or uncertainties.

*I’m kind of looking more towards independence than I previously did… although I might be mentally*,* probably ready for it*,* physically*,* I probably am not… it’s more of a sort of mismatch… I might want it in my head*,* but I’m probably not ready for it. So*,* therefore*,* I don’t want to jump yet*,* but I’m- I’m moving more that way.* [P10]

Most participants voiced uncertainty about their ability to live independently due to concerns about financial stability. They questioned whether they would have the confidence to manage on their own, particularly as they anticipated that living independently of their parents would not afford them the opportunity to save money. The need to balance personal aspirations and external constraints had to be factored into decision-making surrounding the pursuit of independence. Participants described the dichotomy of independence as either a *“necessity”* [P9] or a *“choice”* [P9], emphasising that becoming independent can be shaped by social circumstances and access to basic resources, or by personal preferences, deliberate decision-making, and individual capabilities. Crucially, what drove participants to move towards independence played an important part in how they understood it (sub-theme There are Unseen Dimensions to Independence).

#### There are Unseen Dimensions to Independence

*To be independent*,* I need the multiple variables [pause] to be- in line. Um- [pause-tutting sound] and everything*,* latency*,* duracity*,* time*,* to be also in line. (…….) Independence as a sense of [pause]*,* functionality and that functionality that we talk about (………) functional importance. But let’s not miss the major point in the room. This is where [a] concept comes into play to have functional importance and functionality*,* you firstly*,* need to have psychological functionality. Independence coincides with psychological wellness. If I’m not psychologically well*,* am I going to feel like I’m gonna be able to independently do everything?* [P2]

The complexity and variability of what becoming independent means for autistic people are often underestimated, where the focus is on the *“functional importance”* [P2] (activities of daily living). Our participants spoke about independence as multi-dimensional, encompassing emotional, cognitive, social and psychological dimensions of independence. Some of these aspects were associated with autistic or alexithymic characteristics; many of our participants reported difficulties with independence stemming from challenges in recognising, expressing or regulating emotions, and a few described sensitivities to injustice and conflict. Participants highlighted situations in which emotions had influenced their thoughts, attitudes, or behaviours unintentionally. They also described rumination, difficulty pushing emotions aside, self-blaming for a perceived lack of resilience, and delayed emotional processing. All these experiences presented significant challenges which could compromise independence and yet they were largely invisible.

*You know*,* for the most part*,* and probably outwardly*,* to other people*,* I would seem very independent and capable of doing everything on my own. But I think the- the level of that independence varies from day to day*,* um*,* dependent on all of those other factors*,* um*,* for sure. Um ‘cause you know*,* I definitely notice my ability to be able to do things on my own*,* definitely slips when I’m not doing well (…) um my emotions. So*,* if I’m feeling [little pause] lower*,* I am able to do less.* [P5]

The unseen dimensions of independence, described above, may be unrecognised by outsiders, such as families and social care professionals, who regularly focus purely on functional independence (can someone wash, dress, prepare meals, manage money, travel independently, work).

### Theme 2: “Being Autistic has its Setbacks” in a Neurotypical World

This theme explores how autistic people’s independence is affected by the challenges and disadvantages associated with being autistic. Our participants explained how being *“perceived as different”* [P12] impacts acceptance and understanding. For several participants, *“being autistic has its setbacks”* [P3, P8] in a neurotypical society, not designed to meet their needs, compromised their well-being and independence, due to the lack of understanding and accommodation of core autistic traits. Associated obstacles and limitations restricted their independence in everyday life. Examples participants gave included difficulty accessing community resources such as healthcare settings and supermarkets, and the struggle to maintain personal and working relationships amidst the confusion of unwritten rules.

#### Impact of Mental Health, Trauma and Abuse

Despite participants with severe and enduring mental health problems being ineligible, participants still experienced a range of mental health challenges, including anxiety, depression, obsessive compulsive disorder (OCD), and complex post traumatic stress disorder (PTSD). Several of them also mentioned suicidal thoughts, which they associated with their ability to live independently.

*My mental health*,* ties into my um- independence. If I’m having really bad mental health issues at the time*,* my independence will take a knock*,* I won’t do as many normal things as I would. I won’t- I’ll struggle to sleep at night*,* so therefore get up late for work*,* and I’ll be stressed.* [P12]

Most participants highlighted the pervasive and cyclical nature of the challenges that they faced due to *“being autistic”* [P3, P4, P8, P9], such as being subjected to repeated bullying, abuse, manipulation, and hurt by people in their lives (family, friends, partners, colleagues, acquaintances). This often went unnoticed or unaddressed by people surrounding them and took a huge toll on their mental health and independence.

*I was bullied at school because I didn’t fit in. I was also bullied because I was a geek. I was what they call a dweeb. I- I actually worked hard*,* um- but I was bullied so badly by the time I got to A levels that I stopped- I stopped working. I stopped going to school*,* and I messed up my A levels. And I went back to school at 30.* [P4]

Compromised mental health often had a significant impact on participants’ independence, affecting their ability to perform daily tasks and engage in activities they enjoyed.

#### Lack of Accommodation of Core Autistic Traits

Participants highlighted the fluid nature of independence, which varied from day to day depending on mental health, stressors, and life circumstances such as living arrangements, relationship status, and work pressures. A deterioration in mental health often resulted in disruption of routines that provided predictability, creating a vicious cycle of chaos. Most participants reported constant and intense struggles in managing the demands of daily life, including decoding confusing neurotypical communication and behaviour, and lack of appropriate accommodations for their needs, including sensory sensitivities. All these challenges contributed to the feeling that the world was hostile and unwelcoming to autistic people, and potentially overwhelming, which made it difficult for them to be fully themselves and hindered their ability to live independently.

*They were resurfacing the roads nearby*,* one day*,* and it meant that they had these uh- rollers going on*,* and they had*,* like these jacks on the road*,* so the- the big roller sort of also vibrates*,* but it created really sort of high pitch do-do-do-do-do sort of noise*,* and er I just couldn’t cope with the noise. Basically*,* I had to leave my house. Sort of*,* I- which- which*,* in terms of independent living*,* is- I had to then go somewhere else because of the noise factor (….) and it was just like*,* I cannot live independently here because of the noise [smiles] I have got to go somewhere where there is not noise. Um*,* so then I had to go to my parents’ house.* [P7]

The effort required to navigate a world that did not always understand or accommodate neurodivergence meant that many participants described feeling constantly on the verge of becoming mentally and physically drained, which put their independence at risk.

#### Living on the Brink of Exhaustion and Overwhelm

Participants reflected on difficulties they faced in matching their natural communication style with neurotypical social expectations, to maintain personal and professional relationships. Participants also made frequent references to being overwhelmed by trying to socialise appropriately (as defined by neurotypical standards) which eventually contributed to *“burnout”* [P2] and played a critical role in undermining their independence.

*I was having m-a-j-o-r struggles- in my job role and communication*,* sensory sensitivities*,* um- extreme stress. Um- [pause*,* tutting sound] um- interpretation of language wrong*,* interpretation of actions wrong. What I deemed appropriate wasn’t particularly appropriate. Um- although the intention was honourable*,* the appropriateness was perceived not being honourable.* [P2]

Participants described how they found it challenging trying to navigate social norms and build relationships independently in social or organi sational contexts, as demonstrated by P2 (above). Several participants reported social anxiety about their perceived *“poor social interaction”* [P11] skills which made them *“worried about being misunderstood”* [P4]. They talked about how they struggled to see the hidden meanings or intentions behind the words people were saying and could be misinterpreted themselves. It was clear from these accounts that the constant sensory overwhelm, frequent mutual misinterpretations, and the need to combat and manage stress on a daily basis were undeniably *“exhausting”* [P4, P8, P9] and took a toll on participants’ ability to function independently. As a result, they often found themselves living on the brink of exhaustion and overwhelm.

Several participants attributed challenges in developing daily living skills to a lack of early learning opportunities; in some cases, they thought that they had been exempted from doing chores in childhood because they were autistic. Participants believed that earlier exposure to daily living skills would have enhanced their capabilities and made them more independent. One participant stated that, “*people are just very reluctant to give autistic people their independence*,* because*,* um*,* they’re autistic*,* and they react to certain situations*,* uh*,* very adversely*,* to you know*,* other- children*,* you know. I’ve- I had a lot of moments where I’d panic and meltdown and cry as a kid*,* and*,* um*,* I was a little bit sheltered*,* uh because of that”* [P8].

Structural and systemic factors such as reduced parental expectations, societal reluctance to recognise autistic people’s capabilities, and a lack of appropriate support created barriers to independence and autonomy which sometimes seemed insurmountable to participants.

### Theme 3: Finding Ways of Making it Work

This theme underscores how participants found ways of navigating challenges and striving for independence, despite the challenges posed by being autistic in a predominantly neurotypical society and often having co-occurring mental health conditions. Participants talked about their experiences of four main approaches they took to try to achieve or maintain their desired level of independence: (i) getting support with their mental health and well-being, (ii) not being their authentic autistic self in order to be accepted by neurotypical society, (iii) advocating for their rights and raising awareness of autism, and (iv) *“avoiding throwing away the good with the bad”* [P11] (trying not to make impulsive decisions when things got too difficult to cope with). Through employing these strategies, participants strived to carve out paths that enabled them to lead fulfilling and independent lives despite the obstacles they encountered.

#### Not Going it Alone

Many participants emphasised the importance of mental health support in maintaining their desired level of independence, acknowledging the supportive role of friends, family, and professionals.

*Since I was 12*,* I’ve been having counselling on and off. So it’s something I think I’m gonna have to have throughout the rest of my life*,* (….) Um*,* because*,* I don’t know*,* I guess when I finished counselling*,* I’m kind of like*,* I’m refreshed. I feel great again. You know*,* I’m back to myself*,* um*,* but like*,* I think*,* you know*,* me*,* myself*,* is someone who just needs that*,* ‘cause that is just the support- support I will need continuously throughout my life. Because*,* uh*,* emotions are difficult.* [P8]

Formal or informal mental health support was generally not seen as a *“quick fix”* [P2], but as a lifelong maintenance requirement to offset periods of intense emotional stress and enable them to sustain their desired level of independence.

*I think*,* having a good group of people is important*,* um*,* in terms of support…. I think it’d be nice to have more support*,* but I think you can still be independent through that. Um*,* I think uh distinction for independent living is when you have people come in to do things for you. Um*,* and less less so out of ah choice*,* but more because you can’t do them.* [P7]

Several participants were taking proactive steps to address their individual needs and maintain their mental health and well-being through counselling and therapy. This provided a safe space for them to explore emotions, identify triggers, and develop healthier ways of living that enabled them to thrive, thereby helping them live more independently.

#### Camouflaging as a Survival Technique

Participants, for the most part, felt forced into using masking, camouflaging, or compensating strategies to appear less *“different”* [P12] from neuro-typical people, which they saw as a key factor in fitting into society and thereby gaining independence. Participants shared lots of examples of effortful tactics designed to improve communication, such as preparing planned scripts in advance rather than extemporising when interacting with non-autistic people.

*I’m acting all the time and then I found out what masking was*,* and I was like*,* oh… this is just another reason to make me re- realise that I am autistic*,* ah. Um*,* so yeah*,* I*,* you know*,* I’ve always kind of seen it as a bit of a show. And*,* so others would say*,* oh*,* you know*,* you are really chatty*,* you are like charming*,* and things like that*,* and it’s just like*,* whilst*,* that might be the case to them*,* that’s a LOT of effort for me*,* you know. It doesn’t come easily. I’m not doing it- it’s not natural. Um*,* I feel like I have to put a lot of energy and effort into being*,* you know; being able to socialise in the way that everyone else is.* [P5]

As participants evidenced, failure to conform to social norms around *“greeting with hugs”* [P9] and engaging in *“small talk”* [P3, P5, P6] can result in autistic people being labelled as *“rude”* [P11] and may lead to them being mistreated or even being bullied. This can encourage autistic people mask to appease those around them. However, using scripted social performance is *“effortful”* and *“energy draining”*, as described by P5. Hence the paradox of camouflaging for our participants was that while it made them feel like they were fitting in better in neurotypical society which promoted independence in the short term, it often made things more difficult in the longer term due to the amount of effort involved and the risk of burn out. The lure of adapting behaviour to be accepted by neurotypical people was very strong even though this risked compromising authentic identity, well-being, and independence eventually.

#### Building Bridges of Empowerment & Understanding

Participants emphasised the importance of educating wider society about autism, dispelling misconceptions, and advocating for autistic inclusion to increase autistic people’s independence. Participants described how people would often *“jump to conclusions” [P9]*, expressing scepticism about their professional capabilities if they had visible autistic traits, as autism was stigmatised and assumed to preclude career-based competence. The reaction to someone autistic holding a responsible professional role was often surprising, *“You’re on the spectrum and you work on the [railway] station? I was like*,* yeah. They went*,* what? Bloody hell!”* [P12] Many participants, including P12, reflected on the societal stereotypes or misconceptions about autistic people and their capabilities, highlighting the need for greater awareness and understanding.

*Whenever*,* like*,* people [pause] know that you’re autistic*,* they jump to conclusions. And they think that you can’t do all this other stuff*,* like- and at one of my groups*,* one of the other support people were like- um*,* she was- she was like*,* what*,* you’re an actual teacher?…. And she couldn’t believe that I was an actual teacher. I was like*,* yes I am*,* an actual teacher. (…) Because they see the autistic side.* [P9]

Societal expectations of autistic people are often unfairly low, due to ableism, stigma, and discriminatory structural processes (e.g. recruitment practices), which limits opportunities for independence and self-confidence.

Participants felt that autistic people were often denied opportunities to become independent because of limitations assumed to be intrinsically linked with autistic characteristics rather than situationally specific.

*I think a lot of autistic people in general can struggle a lot because they’re almost not given the opportunity to be independent. I think people are just very reluctant to give autistic people their independence*,* because*,* um*,* they’re autistic*,* and they react to certain situations*,* uh*,* very adversely. Because I think that’s one thing*,* a lot of autistic people struggle with*,* is having this bridge between autistic people and non-autistic people.* [P8]

If autistic participants reacted adversely to situations, this was seen to justify restricting opportunities further rather than addressing contextual and environmental issues. The fit between person and environment was seen to be a key determinant of independence by participants.

#### Avoiding Throwing away the Good with the Bad

Recognising the external challenges that people must overcome, participants explored internal characteristics that act as barriers to independence. Several participants recognised their tendency to dwell on negative emotions which made it difficult for them to recognise their strengths and achieve success. Through the interviews, participants highlighted how someone’s mindset can influence their sense of self and level of confidence, which in turn impacted their ability to live independently.

*I dwell a lot on my emotions [smiles]*,* on my negative emotions. I- I love to sit and ruminate on bad feelings*,* which I don’t think is a good thing. Um*,* I just know I do it a lot. I think I’m a lot more resilient than I give myself credit for. I’d like- I- I think I’m a mess all the time. I feel like life never gives me a break.* [P8]

Participants also sometimes believed that they did not give themselves enough credit for coping in difficult circumstances where one challenge followed on from another. Subjectively they felt like a mess, emphasising a negative outlook, but objectively they realised that their resilience in battling with negative emotions was helping them to attain or regain their independence. This was explicitly stated by one participant, *it’s very easy to make your life worse. It’s very hard to make it better sometimes. So*,* I just keep thinking where instead of just saying all this is rubbish*,* I’m just going to throw it all away. Don’t*,* because you still have something. You know*,* it may not be what you once hoped it was*,* but you still got something* [P11].

Moreover, when asked about what they can do to be more independent when life is generally hard, some participants recognised that they risked impulsively abandoning valuable assets or chasing after things they did not really want or need because of the pressure they were under. Some participants reflected that by avoiding impulsive decisions during difficult times, they could exercise agency and self-control over their actions. This deliberate approach allowed them to navigate adversity while preserving the aspects of their lives that contributed to their overall well-being and independence.

Overall, these themes and sub-themes provide a nuanced and comprehensive understanding of the complex nature of independence as experienced by autistic adults. They highlight both the empowering and challenging dimensions of this pursuit. *Theme 1*,* Independence is “Not a one-size-fits-all”*, reflects the participants’ views that independence can be both a necessity and a choice, with many unseen and personal dimensions shaping their experiences. *Theme 2*,* “Being autistic has its setbacks” in a neurotypical world*, illustrates the profound impact that mental health, trauma, and the lack of accommodations for core autistic traits have on their ability to achieve independence. Finally, *Theme 3*,* Finding ways of making it work*, underscores the importance of support systems, emphasising that achieving independence is not always a solitary journey, but often involves help from and collaboration with others. Together, these themes capture the multifaceted reality of navigating independence as an autistic people.

## Discussion

This study presents the first qualitative data on how autistic people define and experience their independence. Previous research has typically examined independence from a functional perspective (Al Ansari et al., 2024; Ghanouni et al., 2021), taking neurotypical standards and norms as a starting point. In this study, participants were given the freedom to define independence in their own terms, offering a broader and more nuanced perspective. This approach represents a significant shift, as it has provided insights into the more individualised understanding of independence, tailored to the unique experiences of autistic adults. The findings indicated that the autistic adults’ lived experiences of independence involved three main constituents: *Independence is “Not a one-size-fits-all”* (Theme 1), *“Being autistic has its setbacks” in a neurotypical world* (Theme 2) and *Finding ways of making it work* (Theme 3).

Independence was seen by participants as a multi-faceted concept, shaped by various factors such as context, circumstances, and personal choices (Theme 1). Participants’ narratives revealed an understanding of independence that extends beyond mere functional aspects, encompassing emotional, cognitive, social, and psychological domains. A key insight from the study is the inclusion of subtle, unseen dimensions, where independence is influenced by the complex interplay between participants’ internal readiness and external limitations. These unseen dimensions, particularly the desire for psychological independence such as their need for emotional self-sufficiency, and their need for making life choices play a pivotal role in shaping the experience of independence for autistic adults. This perspective contrasts with previous literature, which tends to present independence from a functional lens (Baker et al., 2021; Hume et al., 2014; Spriggs et al., 2017). While functional independence is important, as evidenced by a previous study where participants shared their experiences with moving out and establishing their own living arrangements (Al Ansari et al., 2024), findings from this study suggest that independence is not solely about practical abilities (Wise et al., 2020), but also involves a complex interplay of psychological, emotional, social and cognitive factors. Hence, discussions of independence within the context of autism should not be framed solely through the lens of self-sufficiency but should consider the unique support needs and preferences of autistic people.

Participants described a range of challenges that could get in the way of independence (Theme 2). Noteworthy among the findings are the role of environmental and systemic barriers such as societal norms and expectations, lack of early learning opportunities, and societal reluctance to recognise autistic capabilities in determining the well-being and independence of autistic people. These findings are in line with previous studies, which suggest that societal reluctance to recognize and accommodate the capabilities of autistic people contributes significantly to their marginalisation (Botha, 2020; Crompton et al., 2020; Turnock et al., 2022) leading to internalised ableism. This internalised ableism, as highlighted by Botha et al., (2022), can arise from constant exposure to societal attitudes that regard autism as a flaw rather than a difference. This perception of being flawed, rather than different is echoed in the experiences of many participants, who reported significant challenges in navigating environments. These challenges included difficulties with their mental health and well-being (Black et al., 2024), hindering their access to community resources (Mitter et al., 2019; Zuckerman et al., 2018), personal relationships (Turnock et al., 2022), and employment opportunities (Doyle et al., 2022; Harmuth et al., 2018).

Previous definitions of independence from a functional perspective (Al Ansari et al., 2024; Cribb et al., 2019; Grove et al., 2023) fail to acknowledge the key role of mental health our autistic participants described. Though participants defined independence in a variety of ways, mental health almost always played a significant role in shaping the experience and ability to achieve it. Participants considered that their ability to perform daily tasks and engage in activities they enjoyed were affected by their mental health, in line with previous literature (Hume et al., 2014; Lai et al., 2019).

When prompted about barriers to independent functioning, participants frequently mentioned difficulty managing social situations, including initiating and sustaining interactions, experiencing social anxiety, fear of being misunderstood, and struggles with interpreting social cues. However, this traditional understanding of communication and interaction deficits solely residing within autistic people has been a subject of critique, such as through the conceptualisation of the “double empathy problem” (Milton, 2012, p. 883; Milton et al., 2023, p. 78). Instead of framing autistic social-communication differences solely as deficits within the autistic individual, these challenges may also arise from a lack of understanding and accommodation by neurotypical people. Hence, the difficulties in social interactions are bidirectional (Crompton et al., 2020; Mitchell et al., 2021). Our participants echoed this, explaining how being perceived as different in a neurotypical society negatively affected their well-being and independence. These findings demonstrate that the barriers to independence for autistic adults extend beyond internal challenges and are deeply influenced by a lack of acceptance of core autistic traits, as is evident in Theme 2. This may also be notable in the examples participants gave, which often highlighted barriers to participating independently within social domains.

Participants highlighted the importance of promoting acceptance, support, and accommodation of autism in overcoming barriers to independence and well-being (Theme 3). Many of the challenges participants described were closely tied to barriers in social participation, such as difficulties forming and maintaining relationships, accessing community spaces, and navigating social norms, as reflected in their quotes. This aligns with previous studies emphasising the need for societal changes to facilitate the integration, inclusiveness, and acceptance of autistic people (Kapp, 2018; McMaughan et al., 2024). However, participants also highlighted other barriers to independence, such as sensory challenges in public spaces and limited availability of tailored services. Rather than being a pre-determined focus of the study, the prominence of social participation barriers seemed to reflect how participants themselves conceptualised independence and the restrictions they faced. Previous studies have also supported the notion that achieving full independence, or being self-sufficient with minimal (or no) support is an impossible standard for anyone, including autistic people (Brown, 2012; Pellicano & Heyworth, 2023). Several research studies have shown the effectiveness of therapy or counselling in supporting autistic people with their mental health (Chan & Doran, 2024; Cooper et al., 2017), consistent with the findings of this study. Participants reported that therapy or counselling helped them maintain emotional equilibrium and cope with crises. However, participants in the study also reported barriers to access counselling or support services. The most reported barriers to accessing services were the limited number of autistic-friendly healthcare providers, long waiting times for specialist assessments, and a lack of tailored mental health services (D. Adams & Young, 2021). Additionally, many participants noted a shortage of professionals trained to understand and accommodate the sensory, communication, and social needs of autistic people (D. Adams & Young, 2021). Participants who did not receive adequate support from available services often reported having more unmet healthcare needs. This lack of support was felt to impact their independence negatively in the long term. Moreover, many participants described employing masking or camouflaging techniques to fit into neurotypical society, often at the expense of their authentic selves. There are significant psychological costs associated with masking (Evans et al., 2024), in line with this, participants in the present study reported that masking adversely affected their mental health. Masking was understood to hinder participants’ pursuit of independence by limiting their ability to express their true selves and make authentic choices. Our data are consistent with a view that autistic people would benefit from greater societal acceptance to reduce the pressure on autistic people to conform (Khudiakova et al., 2024; Miller et al., 2021; Pearson & Rose, 2021).

Participants’ accounts revealed the pervasive influence of negative societal stereotypes of autistic people (Botha, 2020; Botha et al., 2022a; Turnock et al., 2022), which often led to feelings of lack of acceptance and understanding reinforcing the need for greater societal awareness and inclusion. Autistic empowerment was identified as a significant factor in participants’ descriptions of their independence, underscoring the importance of feeling capable and valued in society. Moreover, it has been reflected in participants’ narratives that their mental health could have benefited from understanding their experiences through the neurodiversity paradigm, rather than perceiving themselves as inherently flawed (Shaw et al., 2022). To address these misconceptions, it is important that autistic people feel able to self-advocate and empower themselves, and that society becomes better educated about autism and removes barriers of misunderstanding and prejudice that limit access to employment and citizenship (Botha, 2020; Burke et al., 2024). Overall, the interviews illustrated the nuanced relationship between independence and support, highlighting the importance of striking a balance aligned with everyone’s unique needs, circumstances, strengths, capabilities, and aspirations.

### Limitations

While qualitative research does not aim to produce generalisable findings (Braun & Clarke, 2019), it is important to note the participants were not recruited randomly (invitations were sent to an autistic research database and posted via local charities). Therefore, the findings may not fully capture the specific contexts and experiences of the broader autistic community (Johnson et al., 2020). Autistic people vary significantly in terms of their characteristics, needs, and experiences (Wozniak et al., 2017), hence we need multiple studies in future research to capture a wide range of perspectives from autistic adults. Participants reported that if the interview schedule had been shared with them before the interview, it would have helped them provide more insightful answers as they would have had time to recall incidents in advance (Kaplan-Kahn & Caplan, 2023).

In line with our research questions, participants were not given a singular definition of independence as a starting point. In line with previous theorising in the field, over-lapping constructs such as autonomy, self-determination, and empowerment were considered by participants (Graber, 2017; Späth & Jongsma, 2020). Such conceptual fluidity allowed us to identify the sheer breadth of factors that our participants associated with independence. Future studies, however, may look to develop increased conceptual clarity in understanding the independence of autistic people, the independence of neurotypical people, and how they differ.

Although autistic co-researchers played an active role in the later stages of the research (from data analysis onwards), they were recruited too late to influence the initial research design and data collection. Earlier involvement of autistic collaborators would have made the research more inclusive and robust (Hobson et al., 2023; Kaplan-Kahn & Caplan, 2023; Stark et al., 2021). Although we focussed on our participants’ definitions of independence, our research team were likely influenced unconsciously by dominant neuronormative cultural definitions of independence. Though the research team included diverse perspectives, by definition, understanding marginalised perspectives presents additional challenges.

## Conclusions

This is the first study to have focussed on how autistic people conceptualise and experience independence, free from neurotypical assumptions of what independence is. The findings of this study highlight how significant independence is to autistic adults and the value they attach to being able to make choices and exert control. Furthermore, this research sheds light on the challenges and obstacles that autistic adults encounter in their efforts to achieve and maintain independence, identifying areas for support that they consider to be important. Notably, many participants reflected on effortful strategies they used to try to overcome barriers to independence, such as scripting social interactions in advance, camouflaging autistic traits, and building bridges of understanding with non-autistic people to fit in. These findings emphasise the need to ensure that autistic people are understood, supported, and included in society and given opportunities to achieve their desired level of independence without having to compromise their authentic autistic identity, natural communication style, or well-being.

## Electronic Supplementary Material

Below is the link to the electronic supplementary material.

Supplementary Material 1

## Footnotes

1 Day-to-day functioning, includes daily living, communication, and socialization abilities (McQuaid et al., 2022).
