# Supplementary material for: Experiencing Independence: Perspectives from Autistic Adults
Source: J Autism Dev Disord. 2025 Apr 3;56(9):3523–38. doi: 10.1007/s10803-025-06812-0 (PMC13427911; doi:10.1007/s10803-025-06812-0)
Supplement: Supplementary file 1 — Supplementary Material 1 [file 10803_2025_6812_MOESM1_ESM.pdf]

Semi-structured Interview Schedule

1) Can you tell me about your everyday life?
   OR
   Can you tell me what a typical day is like for you?

2) How would you define independence in living your day-to-day life?

3) How do you think about your own level of independence?
   OR
   What is it about your day-to-day life that means it's independent?
   OR
   Can you tell me what an independent life would look like?

4) What are the factors you think are necessary for you to lead an independent life?

5) What are the strategies (plans/ approaches) you follow for improving independence in your everyday life?

6) When things go wrong (or any distress, conflict, problem etc.), how do it affect your independence?

7) What are the barriers or hindrance (if any) you think is affecting your independence in everyday life?
   OR
   What are the difficulties you face in experiencing independence in you day to day life?

8) Do you feel that you need support from others in being independent?
   If YES, in what ways are you are asking for support from others?
   How you seek support when you are in distress?

9) Do you have any specific plan in your future to be more independent than you are now?
